# Activation of integrin and ceramide signalling pathways can inhibit the mitogenic effect of insulin-like growth factor I (IGF-I) in human breast cancer cell lines

**DOI:** 10.1038/sj.bjc.6690113

**Published:** 1999-02

**Authors:** C M Perks, Z P Gill, P V Newcomb, J M P Holly

**Affiliations:** Division of Surgery, Department of Hospital Medicine, Bristol Royal Infirmary, Bristol, BS2 8HW, UK

**Keywords:** integrin and ceramide signalling, insulin-like growth factor I-induced mitogenesis, breast cancer

## Abstract

Cell counting, cell cycle analysis and Western immunoblotting were used to examine the effects of non-apoptotic doses of a ceramide analogue, C2, and a synthetic arginine–glycine–aspartic acid (RGD)-containing peptide, RGD, in MCF-7 and T47D cells to determine whether activation of these signalling pathways could alter the mitogenic potential of insulin-like growth factor I (IGF-I). IGF-I alone increased total cell number in both cell lines, associated with a rise in the percentage of cells in the S-phase of the cell cycle and a co-incident increase in cyclin A production. Treatments alone had no effects on cell number or cyclin A production relative to controls. C2 inhibited IGF-I-induced mitogenesis in both lines, whereas RGD was only effective in the T47D line. Despite inhibition of cell proliferation, IGF-I stimulation of cells in S-phase and of cyclin A levels were unaffected; however, an IGF-I-induced increase in cyclin B1 levels was inhibited by 30%. Low-dose induction of integrin and ceramide signalling pathways causes cells to be blocked in S-phase, thereby inhibiting the normal cycle of events associated with the IGF-I-induced mitotic signal. Activating these pathways may not only restrict tumour growth by induction of apoptosis but they may also directly inhibit IGF-I-induced cell proliferation. © 1999 Cancer Research Campaign

Insulin-like growth factors (IGFs) stimulate the growth of many different cell types including human breast cancer cells (De Leon et al, 1990; Quinn et al, 1996). Breast cancer cells overexpress the type I IGF receptor (Papa et al, 1993), and plasma IGF-I concentrations are higher in primary breast cancer patients (Peyrat et al, 1993). Therefore, IGF-I could be important for maintaining tumour growth in the breast.

The growth of normal adherent cell types in vitro requires not only growth factors but also attachment to the extracellular matrix (ECM) (Ingber, 1990). Cell adhesion to the ECM is mediated by integrin receptors. These bind to matrix proteins outside the cell and associate with cytoskeletal proteins within the cell. Several matrix proteins contain the three-amino-acid sequence, arginine–glycine–aspartic acid (RGD), which is specifically recognized by particular integrin receptors (Ruoslahti et al, 1987). Specifically, integrin-dependent signals have been shown to modulate the control of growth (Giancotti and Ruoslahti, 1990) and cell survival (Frisch and Francis, 1994). Maintenance of integrin linkages is essential for cell adhesion. It has been demonstrated previously that disruption of these attachments, via addition of antibodies or peptides, can induce cells to detach from the substratum (Knudson et al, 1981; Hayman et al, 1985) with a resultant induction of programmed cell death. Cells which become transformed or malignant must, therefore, have acquired the ability to undergo anchorage-independent growth (Freedman and Shin, 1974; Tucker et al, 1981).

The plasma membrane is also the site of sphingomyelin hydrolysis, which is now recognized as an important pathway of signal transduction. Ceramide is one product of sphingomyelin hydrolysis and has been implicated as an important mediator of cell death (Obeid et al, 1993). Tumour growth can be restricted by cytokines, chemotherapy and radiotherapy, which induce programmed cell death via ceramide-mediated cytoplasmic signalling.

The cell cycle is composed of four phases, entry into the cycle (G1), which is dependent on the presence of growth factors, the phase after DNA replication (G2), and the mitotic phase (S) which culminates in cell division (M). On closer examination, it is evident that the cell cycle is a very complex process and relies on a number of different components to ensure an ordered completion of cell division (Grana and Reddy, 1995). One such group of components which plays an essential role is the cyclins, which all undergo periodic synthesis and degradation throughout the cell cycle.

In previous studies (Gill et al, 1997*a*, 1997*b*; Perks et al, 1997) in which we have used ceramide and integrin detachment for inducing apoptosis to investigate IGF survival effects, we had observed that at doses below those required to induce programmed cell death there was marked attenuation of IGF-induced mitogenesis. In this study, we have investigated further how activation of ceramide and integrin signalling pathways may affect IGF-I-induced cell proliferation in human breast cancer cells.

### MATERIALS AND METHODS

Recombinant human IGF-I was purchased from GroPep, Adelaide, Australia. The ceramide analogue C2 (D-*erythro*-sphingosine, *N*-acetyl-) and its negative control (D-*erythro*-sphingosine, dihydro-, *N*-Acetyl-) were bought from Calbiochem, and the synthetic RGD-containing peptide (Gly–Arg–Gly–Asp–Thr–Pro) together with its negative control peptide RGE (Arg–Gly–Glu–Ser) were bought from Sigma. All other reagents were purchased from Sigma or Merck unless otherwise stated.

### Cell culture

Human breast cancer cell lines T47D and MCF-7 were purchased from ECACC, Porton Down, Wiltshire, UK, and grown in a humidified 5% carbon dioxide atmosphere at 37°C. The cells were cultured in Dulbecco's modified Eagle medium (DMEM) with glutamax-1 and minimum essential medium Eagle respectively. Both media were supplemented with 10% fetal calf serum (FCS; Advanced Protein Products), penicillin (5000 IU ml$^{-1}$, streptomycin (5 mg ml$^{-1}$) and L-glutamine (2 mM).

### MTT assay

MTT reagent (3-[4,5-dimethylthiazol-2-yl]-2,5-diphenyltetrazolium bromide; Thiazolyl blue) is converted into a coloured water insoluble formazan salt by the metabolic activity of viable cells and can be used as a crude measure of cell viability. Cells were seeded at $5 \times 10^4$ ml$^{-1}$ (150 µl growth medium) in 96-well plates and were allowed to grow for 24 h. Growth medium was replaced with phenol-red-free, serum-free Hepes-buffered DMEM and Ham's nutrient mix F-12 supplemented with sodium bicarbonate (0.12%) bovine serum albumin (0.2 mg ml$^{-1}$) and transferrin (0.01 mg ml$^{-1}$) (SFM; 100 µl) 24 h before dosing. MTT reagent (7.5 mg ml$^{-1}$) in phosphate-buffered saline (PBS) was added to the cells (10 µl per well) and the cultures were incubated for 30 min at 37°C. The reaction was stopped by the addition of acidified triton buffer [0.1 M hydrochloric acid, 10% (v/v) Triton X-100; 50 µl per well] and the tetrazolium crystals were dissolved by mixing on a Titertek plate shaker for 20 min at room temperature. The samples were measured on a Biorad 450 plate reader at test wavelength of 595 nm and a reference wavelength of 650 nm. Results represent the means ± s.e.m. of five wells from one experiment, which is representative of experiments repeated at least three times; results are expressed as a percentage optical density of SFM controls.

### Cell counting

Aliquots of cells (50 µl) were loaded onto a haemocytometer and the total cell number was determined.

### Flow cytometry

Cells were grown to 65% confluency in six-well dishes (Nunclon) and then washed with phosphate-buffered saline (PBS; × 2). The growth medium was replaced with SFM for 24 h. Cells were then treated with IGF-I (100 ng ml$^{-1}$), a synthetic RGD-containing peptide (10 or 15 µg ml$^{-1}$) or ceramide (1 µM) and coincubations of IGF-I with either RGD or C2, and incubated at 37°C for 48 h. All cells were collected, washed in PBS and fixed by the addition of 70% ethanol (1 ml; 30 min). Cells were then pelleted (6500 r.p.m.; 5 min) and washed three times with PBS (6500 r.p.m.; 5 min). The supernatant was removed and the cells were resuspended in reaction buffer (propidium iodide, 0.05 mg ml$^{-1}$; sodium citrate, 0.1%; RNAse A, 0.02 mg ml$^{-1}$; NP-40, 0.3%; pH 8.3) vortexed and incubated at 4°C for 30 min. All cells were then measured on a Facscalibur flow cytometer (Beckton Dickinson) by an argon laser at 488 nm for excitation, and analysed using Cell Quest (Beckton Dickinson) and Multicycle (Phoenix).

### Western immunoblotting

Cells were grown to 65% confluency in T25 flasks (Nunclon) and then washed twice with PBS. The growth medium was replaced with SFM for 24 h, treated as above for 48 h and then lysed on ice for 10 min (1 ml; 10 mM tris-HCl, 5 mM EDTA, 50 mM sodium chloride, 30 mM sodium pyrophosphate, 50 mM sodium fluoride, 100 µM sodium orthovanadate, 1% Triton, 1 mM phenylmethylsulphonyl fluoride; pH 7.6). A protein assay was then performed on the lysates for normalization of protein levels between samples. The proteins were separated by 12% sodium dodecyl sulphate (SDS) polyacrylamide gel electrophoresis and then transferred onto a nylon membrane, which was probed with either anti-human cyclin B1 or anti-human cyclin A (both at 1.5 µg ml$^{-1}$; Pharmingen) overnight. After the removal of excess unbound antibody, anti-mouse $IgG_1$ and IgE antibodies conjugated to peroxidase (1:3000) were added for 1 h. Binding of the peroxidase was visualized by enhanced chemiluminescence according to the manufacturer's instructions (Amersham International). Optical density measurements were determined using a scanning densitometer (Biorad) and analysed using Molecular Analyst software (Biorad).

### Statistical analysis

The data were analysed using the Microsoft Excel 4.0a software package. Significant effects were determined using Student's $t$-test. A statistically significant difference was considered to be present at $P < 0.05$.

## RESULTS

We investigated the metabolic activity of T47D and MCF-7 human breast cancer cell lines in response to RGD and C2. We also used the corresponding negative controls for the RGD and C2, as outlined in the Materials and Methods section, to establish that the effects of these treatments were specific. The MTT assay was used initially as a crude measurement of cell viability, and this showed that the addition of RGD to the T47Ds (Figure 1A) and MCF-7 cells (data not shown) and C2 to the MCF-7s (Figure 1B) and T47Ds (data not shown) induced a dose-dependent decrease in metabolic activity. The control treatments had no effects on the cells.

Flow cytometry was then used to establish whether this decrease in metabolic activity was due to the induction of programmed cell death or apoptosis and the specific doses at which this occurred. Apoptotic cells have a lower DNA content than normal cells and appear as a pre-G1 peak on a DNA cell cycle histogram. These experiments demonstrated that there was no apoptosis occurring at RGD doses up to and including 10 µg ml$^{-1}$ in the T47D cells (Figure 1C) and MCF-7 cells (data not shown) and at C2 doses of 2 µM and below in the MCF-7 (Figure 1D) and T47D cells (data not shown). However, we established that the large reductions in metabolic activity observed in the MTT assays seen at doses of, for example, 100 µg·ml$^{-1}$ RGD in the T47D (Figure 1C) and MCF-7 cells (data not shown) and at C2 doses of 10 µM in the MCF-7 (Figure 1D) and T47D cells (data not shown) were associated with the induction of programmed cell death.

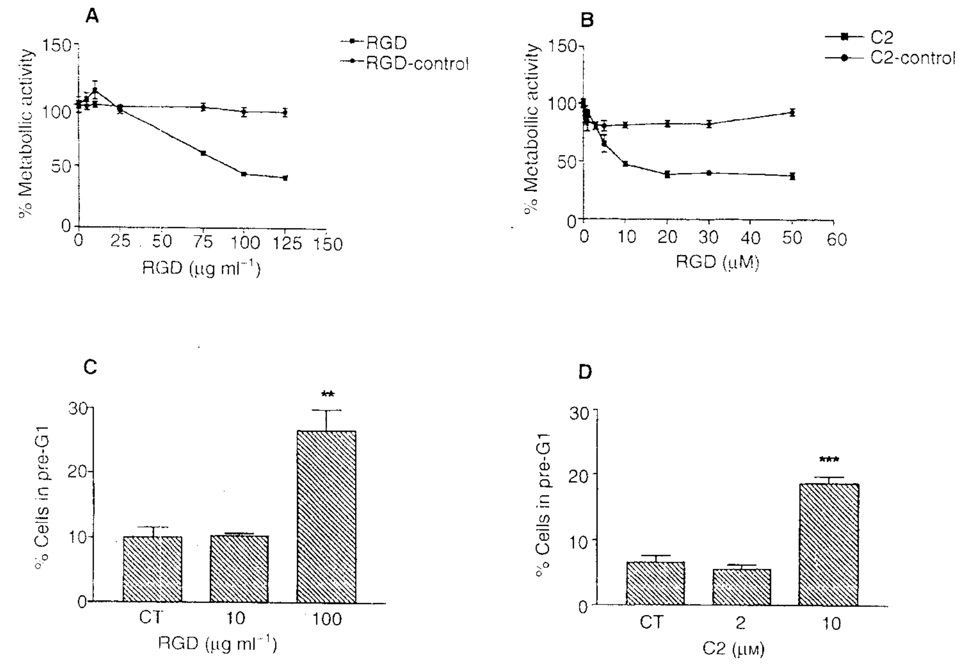

**Figure 1** (**A**) Effects of RGD and RGE (control) (0–125 μg ml⁻¹; in which $P < 0.05$–0.001 at doses of RGD > 25 up to 125 μg ml⁻¹) and (**B**) C2 and dihydro-C2 (control) (0–50 μM; in which $P < 0.05$–0.001 at doses of C2 of 5–50 μM) on the metabolic activity of T47D and MCF-7 cell lines, respectively, after treatment for 48 h. MTT activity was assayed as described in Materials and methods. The results represent the means ± s.e.m. of five wells from one experiment, which is representative of experiments repeated at least three times. (**C** and **D**) Measurement of apoptosis by flow cytometry represents the percentage of cells in the pre-G1 peak of each treated sample in the T47D (0, 10 and 100 μg ml⁻¹) and the MCF-7 (0, 2 and 10 μM) cell lines, respectively, after 48 h treatment. Results represent ± s.e.m. for each dose performed in triplicate simultaneously, and are representative of experiments repeated at least three times (**$P < 0.01$, ***$P < 0.001$)

Having established nonapoptotic doses of these treatments, their effects on the mitogenic potential of IGF-I was then examined. The total cell counts (Figure 2A and B) revealed that IGF-I at 100 ng ml⁻¹ caused a significant ($P < 0.05$, $P < 0.001$) increase in total cell number relative to controls in both the T47D and MCF-7 cell lines respectively. Addition of RGD (10 μg ml⁻¹) alone to T47D (Figure 2A) or MCF-7 (data not shown) cell lines caused no significant differences in cell number in comparison to controls. The addition of RGD (10 μg ml⁻¹) in combination with 100 ng ml⁻¹ IGF-I to the T47D cell line caused a significant ($P < 0.01$) decrease in cell number compared with IGF-I alone (Figure 2A), but this treatment had no effect on IGF-I-induced cell proliferation in the MCF-7 cell line (data not shown). Ceramide analogue C2 (1 μM) alone had no effect on cell number relative to control cells in the MCF-7 (Figure 2B) or T47D (data not shown) cell lines compared with the controls. However, in combination with 100 ng ml⁻¹ IGF-I, C2 (1 μM) caused a significant ($P < 0.01$) decrease in total cell number compared with IGF-I alone in both the MCF-7 (Figure 2B) and T47D (data not shown) cell lines.

Having established that the RGD treatment was only effective in inhibiting IGF-I-induced mitogenesis in the T47Ds, we proceeded to perform all following experiments using RGD specifically in the T47D cells and C2 only in the MCF-7 cells.

The MTT assay further indicated that IGF-I alone caused an approximate 1.6-fold and 2-fold increase in metabolic activity in the T47D and MCF-7 cells respectively. The IGF-I-induced increase in metabolic activity was reduced by approximately 56% with a nonapoptotic dose of 10 μg ml⁻¹ RGD in the T47D cells, and by approximately 35% with 1 μM C2 in the MCF-7 cells (Figure 3A and B).

The cell cycle was then studied to see whether C2 or RGD were inhibiting the entry of cells into the S-phase of the cycle. Results demonstrated in the T47D cells (Figure 4A) that IGF-I alone, as expected, caused a significant ($P < 0.001$) increase in cells in the S-phase of the cycle. IGF-I in combination with RGD still caused a significant ($P < 0.001$) increase in cells in the S-phase. Similarly, in the MCF-7 cells (Figure 4B), IGF-I alone and IGF-I in addition to C2 both produced significant ($P < 0.01$) increases in S-phase.

Figure 5 summarizes the different dose-dependent activities of ceramide and illustrates how IGF-I acts under these conditions. C2 at 0–2 μM has no effect on metabolic activity, but can completely negate the proliferative activity of IGF-I at these doses. C2 at 2–15 μM induces apoptosis in these cells, which can be prevented by the addition of IGF-I at this dose. Furthermore, necrosis occurs at C2 doses of > 15 μM and the cells cannot be rescued from this form of cell death by IGF-I. These data have also been confirmed by cell cycle analysis with flow cytometry (data not shown).

To confirm the cell counting which showed that C2 was inhibiting the mitogenic effect of IGF-I in the MCF-7s, cyclins representative of particular stages of the cell cycle were examined using Western immunoblotting. Cyclins are essential for cell cycle control and they undergo periodic synthesis and degradation. Cyclin A is synthesized during the S-phase and our data (Figure 6) demonstrated that IGF-I in the MCF-7 cells caused a 2.9-fold increase in cyclin A production compared with control cells. This increase was maintained on co-incubation with C2. Cyclin B1 is first synthesized during late S-phase, and is maximally expressed during the transition from G2 to M-phase. Our results showed that cyclin B1 production was increased 2.5-fold compared with control levels by IGF-I alone, but, on co-incubation with C2,

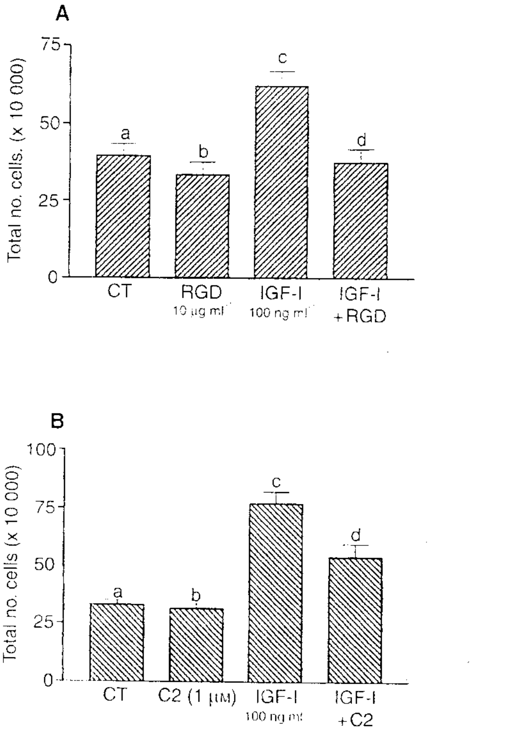

**Figure 2** Measurement of total cell number in T47D and MCF-7 human breast cancer cell lines. (**A**) T47D cells treated with RGD (10 μg $ml^{-1}$), IGF-I (100 ng $ml^{-1}$) or RGD and IGF-I for 48 h in which c > a ($P < 0.05$) and d < c ($P < 0.01$). Results are the means ± s.e.m. of experiments repeated at least three times. (**B**) MCF-7 cells treated with C2 (1 μM), IGF-I (100 ng $ml^{-1}$) or C2 and IGF-I for 48 h in which c > a ($P < 0.001$) and d < c ($P < 0.01$). Results are the means ± s.e.m. of experiments performed at least three times

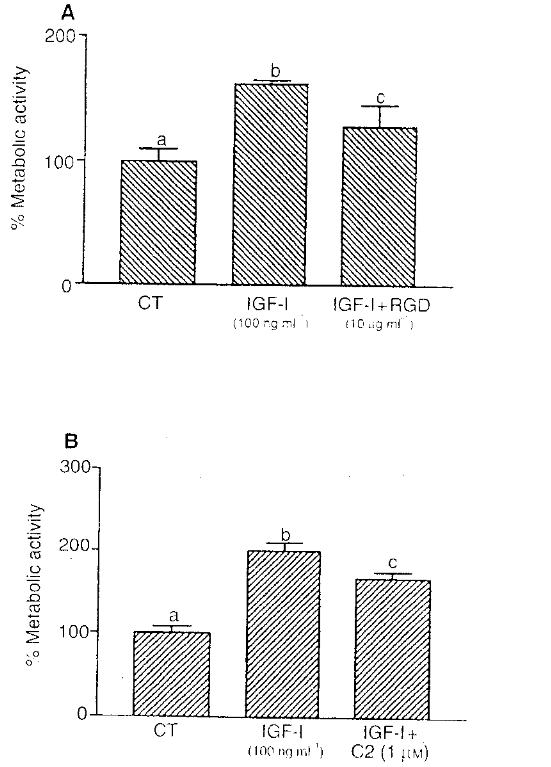

**Figure 3** Effects of RGD and C2 in the T47D and MCF-7 cell lines, respectively, on the mitogenic effects of IGF-I. Results represent cells treated with IGF-I alone (100 ng $ml^{-1}$) and: (**A**) IGF-I in combination with RGD (10 μg $ml^{-1}$) in the T47Ds in which a < b ($P < 0.01$), b > c ($P < 0.05$), and a:c was not significant; (**B**) IGF-I in combination with C2 (1 μM) in the MCF-7 cells in which a < b ($P < 0.01$), a < c ($P < 0.05$), and b > c ($P < 0.05$). MTT activity was assayed as described in Materials and Methods. Results represent ± s.e.m. of five wells from one experiment, which is representative of experiments which have been repeated at least three times

IGF-I-induced cyclin B1 production was decreased by approximately 30%. Preliminary data have shown that comparable changes in cyclin B1 production occurred in the T47D cells after treatment with RGD and IGF-I (data not shown).

### DISCUSSION

Neoplastic growth can result from either a disturbance in the regulation of cell proliferation or in the regulation of cell apoptosis. Since this became apparent, there has been considerable investigation of the factors that regulate apoptosis and their role in controlling tumour growth. Although control of cell proliferation and apoptosis are clearly two distinct processes, in this study we have demonstrated cross-talk between their regulation. Two important mechanisms for controlling apoptosis that have been identified are detachment of integrin receptors from the extracellular matrix and ceramide signalling in response to chemotherapy, radiotherapy and cytokines. We have shown that signalling involved in both of these processes can interfere with the response to an important proliferative growth signal, and that this occurs at levels of signalling below that required to induce apoptosis. This indicates that detachment of integrin receptors and ceramide signalling may restrict tumour growth by affecting both cell proliferation and cell apoptosis.

The activation of the IGF-I receptor by its ligands IGF-I, IGF-II and insulin play a critical role in growth and development (Sara and Hall, 1990; Baserga and Rubin, 1993). In tumour growth, an obvious imbalance occurs with cell proliferation far exceeding programmed cell death. Many malignant tumours, including those in the breast, secrete IGF-I and IGF-II and overexpress the type I IGF receptor (Macaulay, 1992), which suggests that the IGFs may be key players in promoting tumour growth in the breast.

Our results have shown that IGF-I acts as a potent mitogen in both the T47D and MCF-7 cell lines. The increase in total cell number in these cells was accompanied by a corresponding rise in the percentage of cells in the S-phase of the cycle, as anticipated.

Nonapoptotic doses of RGD and C2 in the T47D and MCF-7 cell lines, respectively, had no effect on total cell number compared with controls. The combination of these treatments with IGF-I in the respective cell lines reduced the IGF-I increase in overall cells number. However, cell cycle analysis revealed that the number of cells in the S-phase of the cycle was unaffected by the addition of RGD or C2 compared with IGF-I alone.

Interestingly, we observed that C2 was able to inhibit the mitogenic effect of IGF-I in both the cell lines, but that RGD inhibited IGF-I actions in the T47D but not in the MCF-7 cells. Previous data have demonstrated that ligated integrins transmit specific signals: for example, ectopic expression of $\alpha5\beta1$ integrin, but not $\alpha v\beta1$, suppressed apoptosis of fibronectin-bound Chinese hamster ovary (CHO) cells (Zhang et al, 1995). Our data suggest, therefore, that the T47D and MCF-7 cell lines have distinct complements of integrin receptors and so can respond differently to the same RGD treatment.

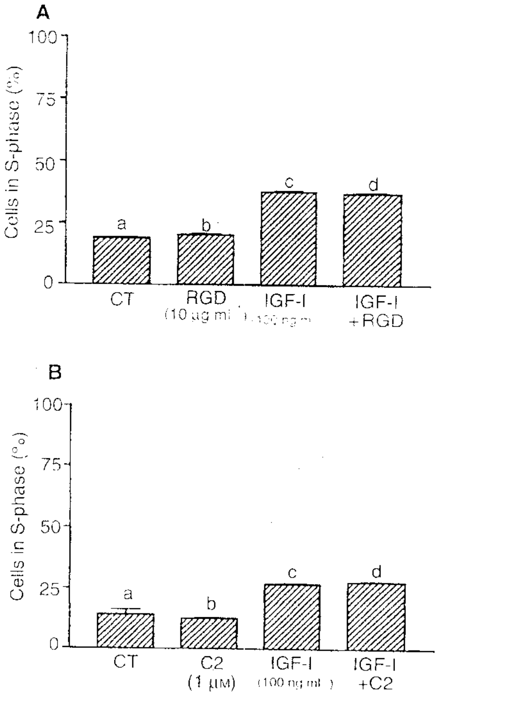

**Figure 4** Flow cytometric analysis of the S-phase of the cell cycle of T47D and MCF-7 cells treated with RGD and C2 respectively. (**A**) T47D human breast cancer cells after treatment with RGD (10 μg ml⁻¹), IGF-I (100 ng ml⁻¹) and RGD and IGF-I for 48 h. Represents the percentage of cells in the S-phase of the cycle (a < c + d, $P < 0.001$; b < c + d, $P < 0.001$; a < b, $P < 0.05$; c > d, $P < 0.05$). (**B**) MCF-7 cells after treatment with C2 (1 μM), IGF-I (100 ng ml⁻¹) or C2 and IGF-I for 48 h. Represents the percentage of cells in the S-phase of the cycle (a < c + d, $P < 0.01$; b < c + d, $P < 0.01$; a:b and c:d, $P > 0.05$). Results are the means ± s.e.m. of experiments performed at least three times

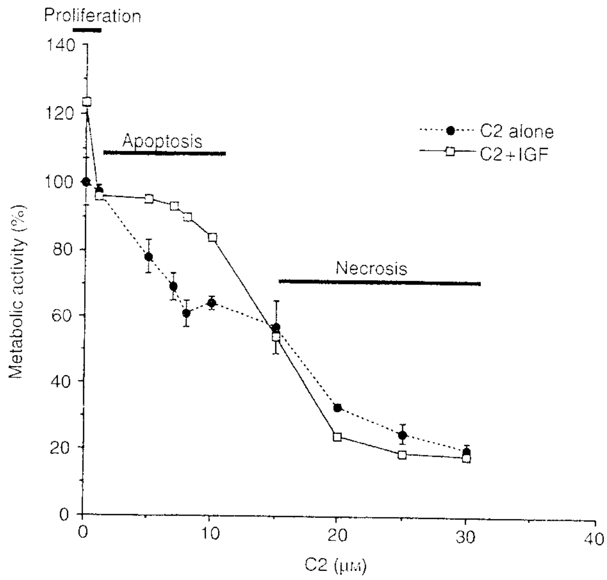

**Figure 5** Represents a dose response to ceramide (0–30 μM) ± IGF-I (100 ng ml⁻¹) for 24 h. MTT activity was assayed as described in Materials and Methods. The results represent the means ± s.e.m. of five wells from one experiment, which is representative of experiments repeated at least three times.

Growth factors are required for cells to enter the cell cycle, but, once past G1 phase, completion of the cell cycle no longer requires mitogens or the matrix (Han et al, 1993). Past this point, there is a switch to intrinsic cell cycle machinery made up of many different components that ensure an ordered progression of cell division (Grana and Reddy, 1995). One such member is the family of cyclins, which play an important role in these events and undergo periodic synthesis and degradation throughout the cycle. We, therefore, looked at cyclin production simply as a marker of cell cycle progression to corroborate our flow cytometry, MTT and cell counting data. Cyclins D1, 2 and 3 are expressed in G1 in response to growth factors, cyclin E is produced later in G1 and peaks at the G1/S transition. S-phase is marked by the expression of cyclin A and this is required for the initiation and progression of DNA replication. Finally, cyclin B1 is maximally up-regulated during the transition from G2/M.

We found, using Western immunoblotting, that in the MCF-7 cells IGF-I alone or in combination with C2 produced equivalent dramatic increases in production of cyclin A compared with control cells. This corresponded to the changes seen previously in the number of cells in the S-phase, as determined by cell cycle analysis. Furthermore, on examining cyclin B1 production, we observed that IGF-I alone caused significant increases in its levels relative to controls, but on co-incubation with C2 these concentrations were reduced. This observation supported the reduction in overall cell number that was seen on co-incubation of the MCF-7 cells with IGF-I and C2 compared with IGF-I alone. This indicated

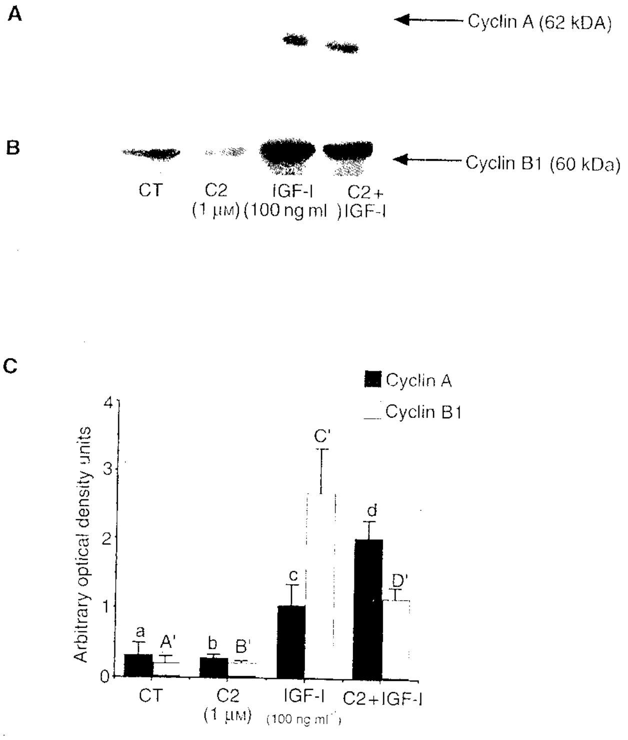

**Figure 6** Measurement of cyclin A and cyclin B1 by Western immunoblotting. Production of cyclins A (60 kDa) and B1 (62 kDa) in MCF-7 cells treated for 48 h with C2 (1 μM), IGF-I alone (100 ng ml⁻¹) or C2 in combination with IGF-I. **A** and **B** are representative immunoblots of cyclin A and B1 respectively. **C** shows the means ± s.e.m. optical density readings from immunoblots performed at least three times, in which for cyclin B1: A′:B′, $P > 0.05$; A′ < C′, $P < 0.01$; A′ < D′, $P < 0.01$; C′ > D′, $P < 0.05$ and for cyclin A: a:b, $P > 0.05$; a < c, $P < 0.01$; b < c, $P < 0.01$; b < d, $P < 0.01$; a < d, $P < 0.001$

that although cells were being pushed into the cell cycle this was not being completed by cell replication.

In summary, these data demonstrate that treatment of MCF-7 and T47D cells with IGF-I alone or in combination with nonapoptotic doses of C2 or RGD, respectively, leads to comparable numbers of cells in the S-phase and equivalent concentrations of cyclin A, whereas there is a reduction in both overall cell proliferation and production of cyclin B1. This analysis suggests that both C2 and RGD at nonapoptotic doses are able to block cells in the S-phase of the cycle, thereby modulating the normal cycle of events usually initiated by IGF-I. Therefore, these nonapoptotic doses of C2 and RGD have the ability to modulate the cell cycle events associated with the mitotic signal induced by IGF-I in these cell lines.

In conclusion, a balance between the rate of cell proliferation and cell death by apoptosis has to exist to ensure tissue homeostasis. A tumour could arise via a combination of inappropriate cell proliferation and reduced cell death by apoptosis. It has been demonstrated previously that cells require attachment to the matrix for normal growth but can be triggered into apoptosis by detachment from the substratum. Malignant cells have, therefore, acquired the ability to undergo anchorage-independent growth, and are no longer susceptible to detachment as the normal trigger for programmed cell death. It has also been demonstrated previously that activation of ceramide signalling pathways via cytokines, radiotherapy and chemotherapy will induce programmed cell death. We have now shown that induction of ceramide and integrin signalling pathways at nonapoptotic doses can also have important consequences in tumour biology. Our data suggest that the response to growth factor stimulation of cells may be modulated by low-dose activation of ceramide and integrin signalling pathways.

### ACKNOWLEDGEMENTS

We wish to thank the Medical Research Council and Zeneca Pharmaceuticals for financial support.
